# T-cell senescence and immunosenescence in chronic oral mucosal inflammatory diseases: mechanisms and therapeutic implications

**DOI:** 10.1080/20002297.2026.2728747

**Published:** 2026-09-27

**Authors:** Huaijue Qiu, Yilin Luo, Lei Pu, Xue Luo, Xiliang Jiang, Moussa Ide Nasser, Chi Liu, Kaidiliya Yalikun

**Affiliations:** a University of Electronic Science and Technology of China, School of Medicine, Chengdu, China; b Department of Stomatology, Sichuan Academy of Medical Sciences· Sichuan Provincial People's Hospital, Chengdu, China; c Department of Nephrology and Institute of Nephrology, Sichuan Provincial People's Hospital; Sichuan Provincial Clinical Research Center for Kidney Diseases; Key Medical Laboratory of Precision Prevention and Treatment of Kidney Diseases; School of Medicine, University of Electronic Science and Technology of China, China; d Department of Cardiac Surgery, Guangdong Provincial People's Hospital (Guangdong Academy of Medical Sciences), Southern Medical University, Guangdong Cardiovascular Institute, Guangzhou, China

**Keywords:** Oral mucosal diseases, T-cell aging, oral lichen planus, Behçet's disease, recurrent aphthous ulcers

## Abstract

**Background:**

The oral mucosa is a dynamic barrier. T‑cell dysregulation contributes to chronic oral mucosal inflammatory diseases, but the role of age‑related immune remodeling remains unclear..

**Method:**

We performed a narrative review of PubMed/Medline (1972–2026) on T‑cell subsets, immunosenescence, exhaustion, and inflammation in oral lichen planus (OLP), Behçet's disease (BD), and recurrent aphthous stomatitis (RAS). After screening, we synthesized 121 publications.

**Results:**

Disease‑specific patterns emerged. In OLP, activated CD8⁺ and tissue‑resident memory T cells associate with basal keratinocyte injury, while senescent mesenchymal cells may amplify inflammation via SASP. In BD, Th1/Th17 polarization, reduced regulatory control, and neutrophil activation drive systemic and mucosal inflammation. In RAS, Th1‑skewed and CD8⁺ responses correlate with epithelial damage. Direct causal evidence for senescent or exhausted T cells is limited, especially for BD and RAS.

**Conclusion:**

T‑cell senescence may modulate disease course, but its effects are context‑dependent rather than universal drivers. The strongest support exists for senescence‑associated stromal signaling in OLP; evidence in BD and RAS is mainly associative. Therapeutic strategies targeting senescence remain hypothesis‑generating and largely preclinical.

## Introduction

Oral mucosal diseases encompass infectious, immune-mediated and ulcerative conditions. Earlier work emphasised microbial infection and mechanical trauma. In contrast, more recent studies have highlighted the role of cellular immunity at a barrier continually exposed to the oral microbiome, mastication, food antigens and environmental stimuli. RAS has been associated with alterations in T-lymphocyte subsets and cellular immunity [1,2], and OLP with aberrant T-cell-mediated responses [3], and BD with systemic immune dysregulation that can include an altered CD4+/CD8+ ratio [4]. Experimental and general immune-aging literature suggests possible links between persistent T-cell stimulation and senescence [5]. However, disease-specific causal evidence in oral mucosal disorders remains limited.

### Methods

This is a narrative review. We searched PubMed and Medline for English-language articles published from 1972 to 2026. Searches combined the following MeSH terms and keywords: ‘T-cell senescence’, ‘immunosenescence’, ‘T-cell exhaustion’, ‘chronic T-cell activation’, ‘senescence-associated secretory phenotype’, ‘oral lichen planus’, ‘Behçet's disease’, ‘recurrent aphthous stomatitis’, ‘tissue-resident memory T cells’, ‘CD4+ T cells’, ‘CD8+ T cells’, ‘regulatory T cells’, ‘Th17 cells’ and ‘mucosal immunity’.

Inclusion criteria were: (1) original human studies examining T-cell subsets or immune mechanisms in OLP, BD, or RAS; (2) animal or in vitro mechanistic studies relevant to T-cell biology in these diseases; and (3) systematic reviews, meta-analyses, or authoritative narrative reviews used for background and conceptual framing. Exclusion criteria were: (1) studies not primarily relevant to T-cell immunity or immune aging; (2) case reports or case series with fewer than five participants; and (3) studies focused on oral malignancy or non-inflammatory lesions.

Duplicates were removed manually. Two reviewers independently screened titles and abstracts and then assessed relevant full texts. Studies were categorised as oral tissue-based human studies, peripheral-blood studies, animal or in vitro models, or review articles. The final reference set comprised 121 publications. Because this was a narrative rather than a systematic review, no formal risk-of-bias or certainty-of-evidence tool was applied, and no quantitative synthesis was performed. We therefore distinguish direct evidence of oral disease from general evidence of immune aging and prioritise larger, better-controlled and mechanistically informative studies when concluding.

### Oral mucosal immune system

The oropharyngeal cavity's complex anatomical structure favours colonisation by various microorganisms. Beyond dynamic symbiotic microbiota, external stimuli, including food particles and their antigens, airborne contaminants and other orally exposed exogenous substances, pose substantial challenges to oral mucosal homoeostasis [6]. The precise mechanisms by which these diverse signals modulate the oral mucosal immune system remain poorly understood. Nevertheless, the immune system of the oral mucosal barrier is adept at adapting to environmental stresses, providing robust protection against pathogenic threats while maintaining tolerance to commensal microbiota and harmless antigens, thereby preventing excessive inflammatory responses [7].

Within the oral cavity, the periodontal epithelium (e.g. gingiva) surrounds the teeth, providing a stable attachment and a sealing interface. Meanwhile, the periodontal epithelium is also a vulnerable site for microbial invasion of the periodontal environment [6]. Consequently, the oral mucosa and the inherent mucosal immune system are critical to maintaining the integrity of the internal milieu. The mucin, lysozyme, secretory immunoglobulin A, lactoferrin and peroxidase in saliva, as well as IgG, IgM, IgA, lysozyme and peroxidase in gingival crevice fluid, play significant roles in regulating mucosal immunity. Moreover, the mucosal immune system mounts responses through the sustained presence of immune cells within mucosal compartments. Regulatory T cells and T lymphocytes that reside in mucosal tissue are integral to both mucosal immunity and immune tolerance [6]. According to the relevant literature, some researchers have demonstrated early on in mouse models that CD4+ T cells are key mediators of the pathology of periodontitis and oral mucosal diseases [8,9] (Figure 1). Both CD4^+^ and CD8+ T cells in mice and humans are dominant, with the majority of CD4^+^ and CD8^+^ T cells exhibiting a memory phenotype [10,11]. The resident effector CD4^+^ and CD8^+^ T-cell populations in the gingiva of mice and humans produce typical type 1 and type 17 effector cytokines, including IFN-*γ* and IL-1, in the gingival fluid [10,11]. Studies have demonstrated that these cells contribute to early and immediate immune defence mechanisms, providing site-specific protection against pathogenic insults [12].

**Figure 1.**
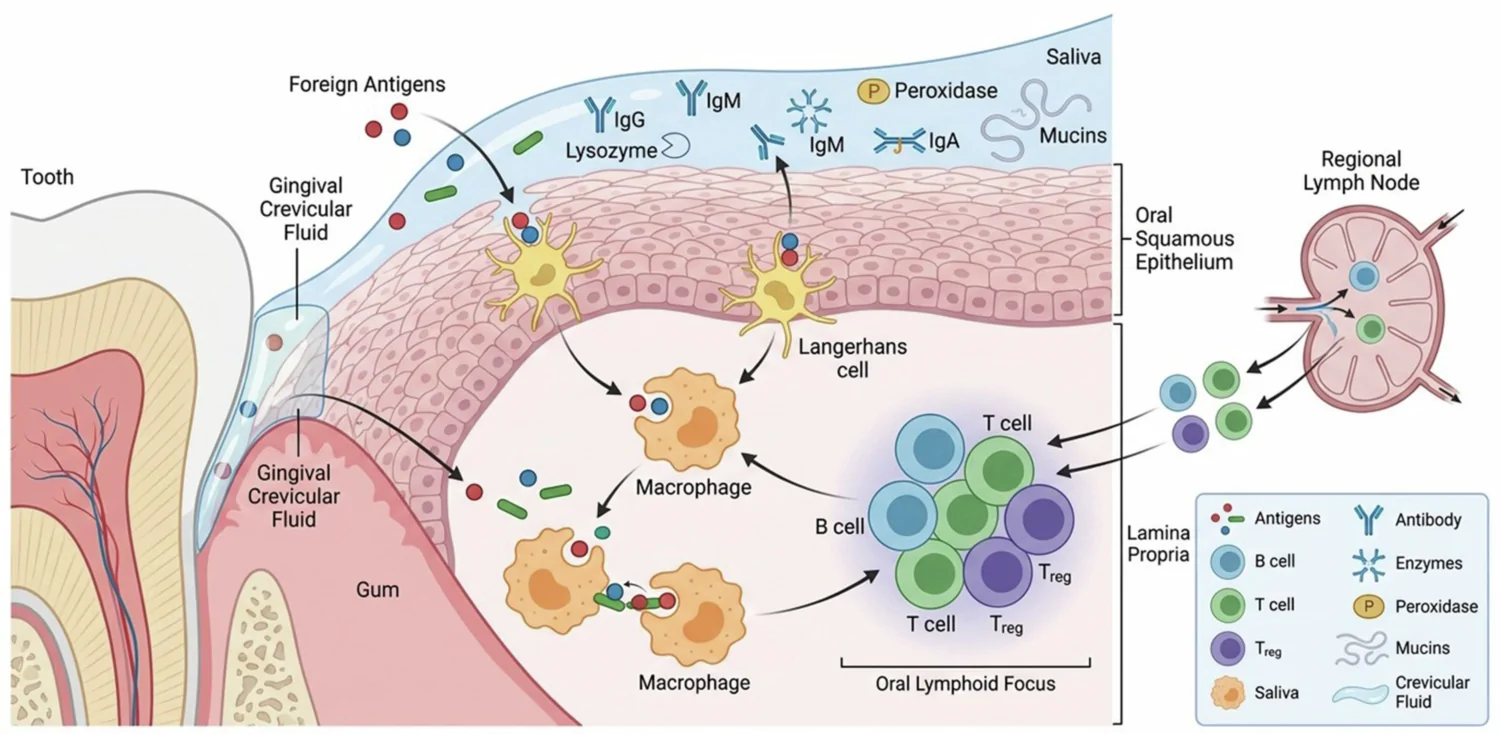
Mechanisms of oral epithelial damage induced by T-cell activation in response to stimuli. This diagram illustrates how external stimuli (viral infection, bacterial products, mechanical trauma) trigger T cell-mediated damage to the oral epithelium: Target cells and Langerhans/antigen-presenting cells present antigens (A1, A2) via MHC-II and MHC-I molecules, respectively, to activate CD8⁺ and CD4⁺ T cells. Activated CD8⁺ T cells secrete IL-2, while CD4⁺ T cells release IFN-γ; together, these cytokines (along with interactions involving RCA/RCAI) drive the induction of oral epithelial damage.

Persistent physiological damage caused by mastication is an important tissue-specific signal at the oral barrier. In mouse models, this mechanical stress promotes homoeostatic Th17 responses that support barrier protection [13]. Studies using mouse models have demonstrated that the accumulation of Th17 cells in the gingiva is a response to physiological barrier damage induced by mastication [13]. Th17 cells have emerged as significant regulatory factors in the homoeostasis of oral barrier tissues and in the pathogenesis of immune-related disorders [14,15]. The physiological role of Th17 cells has been robustly demonstrated in fungal immune surveillance; however, their dysregulation is linked to the periodontal immunopathology of experimental periodontitis [14,16] and to hereditary forms of periodontitis in humans [16]. Additionally, specific research groups have reported an expansion of Th17 cells within the gingival tissue with aging [17]. However, the frequently cited aging studies did not specifically demonstrate expansion of gingival Th17 cells. Instead, immunohistochemical studies in aging mice reported increases in total T lymphocytes in the attachment zone and papillary connective tissue [18]. Together with age-related changes in local immune-cell distribution and tissue susceptibility [19]. Thus, an age-related increase in gingival Th17 cells should not be inferred without direct subset-specific evidence (Figure 2).

**Figure 2.**
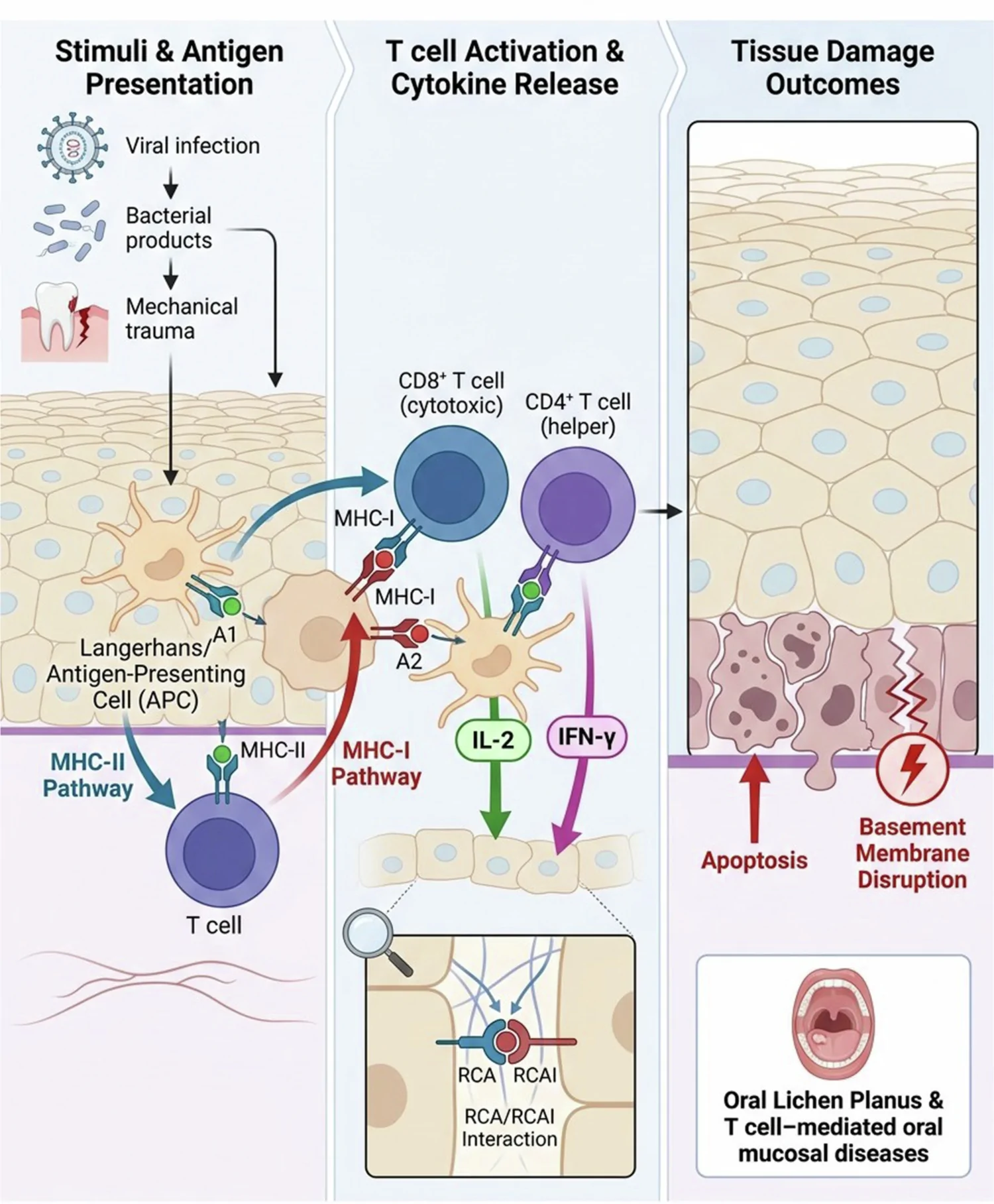
Oral mucosal immune response at the effector site: cellular and humoral components. This diagram depicts the immune defence network at the oral mucosa (effector site, e.g. around a tooth): Foreign antigens interact with immune cells (macrophages, Langerhans cells) in the epithelium and lamina propria; these cells, along with B cells, T cells and T regulatory cells (recruited from regional lymph nodes), form oral lymphoid foci to mediate local immunity. Additionally, oral fluids (gingival crevicular fluid, saliva) contain immune molecules (antibodies such as IgG/IgM/IgA, enzymes and mucins) that support this protective response.

### Distinguishing immunosenescence, T-cell exhaustion and chronic activation

Before examining the role of T cells in specific oral mucosal diseases, it is essential to clarify three interrelated but distinct immunological concepts that are often conflated in the literature: immunosenescence, T-cell exhaustion and chronic activation.

Immunosenescence refers to broad age-associated remodelling of innate and adaptive immunity [20]. Hallmarks include thymic involution, reduced naïve T-cell output, accumulation of memory T cells, loss of T-cell receptor (TCR) repertoire diversity and the emergence of senescence-associated secretory phenotype (SASP) in various cell types [21]. Immunosenescence is a global, age-driven process that affects both innate and adaptive immunity.

T-cell exhaustion, in contrast, is a state of progressive T-cell dysfunction that arises in the context of chronic antigenic stimulation such as persistent viral infections (e.g. CMV, HIV) or tumours [21]. Exhausted T cells are characterised by sustained expression of inhibitory receptors (e.g. PD-1, TIM-3, LAG-3), progressive loss of effector functions (first IL-2, then TNF-*α*, then IFN-*γ*) and altered metabolic and transcriptional programmes [22]. Importantly, exhaustion is reversible to some extent upon blockade of inhibitory pathways (e.g. immune checkpoint inhibitors), whereas immunosenescence is largely irreversible.

Chronic activation refers to persistent T-cell stimulation without resolution. Activated cells may express markers such as CD69, CD38, or HLA-DR and continue to produce inflammatory mediators. Unlike exhaustion, chronic activation does not necessarily imply loss of function; it may instead sustain tissue injury. Activation markers, inhibitory receptors and functional assays are therefore required before classifying a population as activated, exhausted, or senescent.

These three processes are not mutually exclusive. In chronic inflammatory diseases such as OLP, BD and RAS, persistent T-cell activation may accelerate immunosenescence, and senescent T cells may in turn be more susceptible to exhaustion [23]. Understanding these distinctions is critical for interpreting the evidence presented in this review and for developing targeted therapeutic strategies.

### Aging of the adaptive immune system: beyond CD4^+^ T cells and Tregs

With advancing age, naïve T-cell numbers decline, memory populations expand and T-cell receptor diversity contracts. These changes reduce responsiveness to novel antigens and may weaken vaccine and recall responses [24]. Chronic antigen exposure, including persistent viral infection, can further shape the accumulation of highly differentiated or senescence-associated T-cell populations [25]. Changes in CD4+ subsets and regulatory T cells are heterogeneous across individuals and disease contexts; increased Treg frequency does not necessarily imply preserved suppressive function [25–27]. Studies have indicated that functional deficiencies in Tregs are instrumental in the pathogenesis of autoimmune diseases [28]. The quantity and function of naturally occurring Tregs increase with advancing age [29]. This age-related rise in Tregs has been associated with various cancers [30–32] and with impaired immune responses [33,34]. However, current research has yet to clarify whether alterations in Treg function during aging are the definitive cause of the increased prevalence of autoimmune diseases in older adults.

Beyond CD4+ T cells and Tregs, aging profoundly affects CD8+ T cells. The attrition of circulating naïve CD8+ T cells is more pronounced than that of naïve CD4+ T cells in older individuals, as evidenced by both relative and absolute cell counts [35]. Aged CD8+ T cells exhibit diminished proliferative capacity, reduced cytotoxic function and impaired ability to generate robust memory responses following infection [35].

Although natural killer (NK) cells are innate rather than adaptive lymphocytes, they are relevant comparators in mucosal immune aging. Age-related changes in NK-cell phenotype, proliferative capacity and homoeostasis have been reported [35]. Their specific contribution to age-related outcomes in oral mucosal inflammatory disease remains uncertain.

Tissue-resident memory T (Trm) cells represent a recently characterised population of terminally differentiated T cells that reside permanently in peripheral tissues, including the oral mucosa. Trm cells provide rapid local immune responses upon antigen re-encounter. However, their persistence in inflamed tissues may also contribute to chronic inflammation and disease recurrence in autoimmune conditions [35]. The impact of aging on Trm cell formation, maintenance and function in the oral mucosa remains an emerging area of investigation.

### Age-related alterations in T-cell repertoire and function

The T-cell system serves as an exemplary model for investigating the effects of aging on cellular population dynamics. Immune competence is determined by the frequency with which T cells recognise specific antigenic peptides. Studies using experimental mouse models have demonstrated that substantial defects in CD4^+^ and CD8^+^ T-cell responses emerge with advancing age [5]. Research has demonstrated that aged mice [36] are unable to elicit a strong and sustained CD8^+^ T-cell memory response following infection. This inadequacy may stem from deficiencies in the primary immune response, attributable to either functional impairments or diminished diversity within the CD8^+^ T-cell repertoire. Furthermore, intrinsic defects in senescent CD4^+^ T cells restrict initial activation events, significantly impairing B-cell proliferation and differentiation and reducing their ability to provide cognate help [5]. Also, studies have indicated that, similar to CD4^+^ T cells, the responsiveness of CD8^+^ T cells to newly encountered antigens diminishes with advancing age. Notably, the attrition of circulating naïve CD8^+^ T cells is more pronounced in older individuals, as evidenced by both relative and absolute cell counts [37–39]. The absolute and relative numbers of naïve T cells decline progressively with advancing age, likely due to an inability to maintain the homoeostatic proliferation of these immature cells. This decrease is relatively modest for naïve CD4^+^ T cells; however, it is markedly pronounced for naïve CD8^+^ T cells, even in healthy elderly individuals [24]. Aging also significantly influences the generation of CD8^+^ T-cell memory and recall responses in older people. Notably, the effects of thymic involution are closely associated with the age-related acquisition of functional deficits in T cells. As T cells experience senescence in peripheral environments, these impairments accumulate, culminating in a significant reduction in immune competence among older adults [5,24]. In the context of oral mucosal diseases, OLP is defined as a T cell-mediated chronic inflammatory disorder. The pathogenesis of BD is characterised by an imbalance in the Th1/Th2 and Th17/Th1 ratios, along with increased cytotoxic CD8^+^ T cells and reduced Tregs, thereby highlighting the intricate nature of the immune responses involved in BD [40]. The incidence of RAS is similarly associated with peripheral T-cell aging. The immunoregulatory mechanisms underlying these autoimmune oral mucosal diseases are intricately connected to T-cell dynamics. Nevertheless, the effects of immunosenescence on the prevalence and progression of these conditions, as T-cell immunity declines with advancing age, require further scholarly exploration.

### Disease-specific relationships between oral mucosal diseases and T-cell immunity

OLP, BD and RAS were selected because they represent three clinically and immunologically distinct patterns in which T-cell-mediated responses are prominent: chronic epithelial interface inflammation in OLP, systemic vasculitis with recurrent oral involvement in BD and recurrent self-limited ulceration in RAS. They are compared to identify disease-specific, not universal, relationships with immune aging. Autoantibody-dominant diseases such as pemphigus vulgaris and mucous membrane pemphigoid were outside the scope of this review because their primary pathogenic mechanism is humoral. The conclusions drawn from OLP, BD and RAS should therefore not be generalised to all oral mucosal diseases.

### Oral lichen planus (OLP)

OLP is considered the oral manifestation of mucocutaneous lichen planus. As the oral cavity functions as the initial segment of the digestive system and is integral to the gut–skin axis, disruptions in the composition of the oral and salivary microbiota can directly induce immune dysregulation, contributing to the pathogenesis of OLP [41,42]. OLP is observed in approximately 70% of patients with classic cutaneous lichen planus, with oral manifestations being the sole clinical presentation in 20–30% of cases. Although OLP can manifest in six distinct morphological forms, the reticular variant is the most predominant. It is characterised by chronic pain, a persistent and refractory course and carries a potential risk of malignant transformation into squamous cell carcinoma [43,44]. Current understanding suggests that the immunopathogenesis of lichen planus is primarily driven by cell-mediated cytotoxicity, particularly involving cytotoxic T lymphocytes [3]. Cytotoxic T-cell infiltration into the basement membrane zone leads to the apoptosis of basal keratinocytes and subsequent disruption of the basement membrane [45], However, there is also infiltration of varying proportions of B cells, natural killer (NK) cells and macrophages [29]. Most cases of lichen planus are idiopathic, and the aetiology remains unclear. However, cell-mediated immune responses following changes in mucosal antigens are considered to play a significant role in its pathogenesis [46]. Studies have found that increased Langerhans cell presence in OLP suggests that the adaptive immune system may play a crucial role in the pathogenesis of oral lichen planus [47,48]. It is further hypothesised that activated Langerhans cells present an unknown antigen to CD4^+^ T cells, which, through adhesion molecules, triggers CD8^+^ T cell-mediated epithelial destruction [49]. Most T cells associated with OLP are composed of CD4^+^ helper T cells and CD8^+^ cytotoxic T cells. The lymphocytic infiltrate in OLP is predominantly constituted by T cells, with a significant proportion of activated CD8^+^ T lymphocytes localised within the epithelium and in proximity to damaged basal keratinocytes. Importantly, there is no concomitant increase in CD4^+^ T cells within regions exhibiting basement membrane destruction [45,50]. Studies suggest that the activation of cytotoxic CD8^+^ T cells in OLP occurs through the interaction of basal keratinocyte antigens with MHC class I molecules. Additionally, CD4^+^ T cells secrete interleukin-2 (IL-2) and interferon-*γ* (IFN-*γ*), which further promote the apoptosis of keratinocytes induced by cytotoxic CD8^+^ T cells [51–53]. Activated helper T cells secrete IL-2 and IFN-*γ*, which activate cytotoxic T cells and promote their proliferation [54]. Activated cytotoxic T cells can induce apoptosis of basal keratinocytes, leading to liquefactive degeneration of the basal epithelium. This pathological process is frequently observed in OLP lesions and plays a pivotal role in the chronic inflammation characteristic of OLP [55].

Furthermore, research on CD8^+^ tissue-resident memory T (Trm) cells, which secrete a diverse array of cytokines, has elucidated their role in the pathogenesis of OLP, particularly in non-erosive oral lichen planus (NEOLP) and erosive oral lichen planus (EOLP). Tissue-resident Trm cells, a recently characterised population of terminally differentiated T cells, play a critical role in local immune responses [56]. CD8^+^ Trm cells may influence the biological processes of OLP by releasing cytokines such as TNF, IFN and IL-17, suggesting that their presence is closely associated with OLP clinical subtypes [57]. Immunofluorescence studies have confirmed the presence of CD8^+^ Trm cells in both NEOLP and EOLP, with significantly higher abundance in EOLP. Indeed, EOLP exhibits a more pronounced accumulation of CD8^+^ Trm cells beneath the eroded epithelium, and their numbers are significantly elevated compared to NEOLP, with areas of the mucosal epithelial basement membrane appearing indistinct [57]. The excessive release of cytokines, such as IFN-*γ*, may influence the clinical subtype and prognosis of OLP, positioning CD8^+^ Trm cells as significant facilitators of the recurrence of erosive lesions [57]. Investigations into autoimmunity suggest that recurrent exacerbations in autoimmune diseases may be partially attributable to the sustained presence of tissue-resident Trm cells, which are difficult to eradicate [58,59]. Therefore, we might conclude that OLP is classified as a cell-mediated autoimmune disorder, significantly influenced by T lymphocytes, including CD8+ T cells and CD8+ Trm cells, as well as the cytokines they secrete. As the organism ages, the decline in immune competence, especially in T-cell differentiation and senescence, exerts a substantial impact on the pathogenesis of OLP. Nevertheless, the precise interplay between these factors necessitates further exploration.

As well, OLP is a T-cell-mediated chronic inflammatory disorder. Indeed, immunohistochemical analyses have revealed a marked increase in senescent mesenchymal cells in the subepithelial layer of patients with OLP. Employing CellChat for intercellular communication analysis has demonstrated that senescent mesenchymal cells activate and recruit CD8^+^ T cells and NK cells via the CXCL12-CXCR4 signalling axis. Moreover, in vitro studies have shown that senescence-associated factors secreted by mesenchymal cells can stimulate the activation of both T cells and NK cells, thereby promoting epithelial cell senescence and cytotoxicity. These findings indicate that the accumulation of mesenchymal cells with a senescence-associated secretory phenotype (SASP) may be a pivotal driver of OLP pathogenesis. Previous studies have shown that accumulated senescent cells express a SASP, leading to excessive infiltration of immune cells, such as T cells and NK cells, which, in turn, contributes to chronic inflammation. In a study examining the relationship between cellular senescence and the pathogenesis of OLP, an analysis of age-related variations in OLP patients revealed significant enrichment of senescence-associated genes within their genomic profiles. These results support an association between senescence and OLP. The study showed that clusters of senescent mesenchymal cells exhibit robust interactions with immune cells, particularly CD8^+^ T cells and NK cells. Furthermore, it demonstrated that these senescent mesenchymal cell clusters are significantly associated with immune cells via the CXCL12-CXCR4 signalling axis. In this context, CXCL12 functions as a ligand for CXCR4, a receptor instrumental in activating CD8^+^ T cells and NK cells and in mediating their recruitment to cells expressing CXCL12 [60–62]. The research findings indicate that as cellular senescence progresses, enhanced CXCL12-CXCR4 signalling in senescent mesenchymal cells may lead to the accumulation of CD8^+^ T cells and NK cells adjacent to these cells in OLP patients. This accumulation could induce epithelial cell senescence and cytotoxicity, thereby facilitating the pathological changes associated with OLP [63].

In addition to the CXCL12-CXCR4 axis, other chemokine axes have been implicated in T-cell trafficking and activation in OLP lesions [64]. The CCR5-CCL5 axis mediates recruitment of CD8+ T cells and Th1 cells to inflammatory sites [65]. At the same time, CCR7-CCL19/CCL21 is involved in T-cell homing to lymphoid tissues and may regulate the balance between resident and circulating T-cell populations [66]. The CXCR3-CXCL9/10/11 axis, which is critical for Th1 cell recruitment, has also been reported to be upregulated in OLP lesions [67]. Collectively, these findings suggest a complex chemokine network rather than a single dominant pathway in OLP pathogenesis. Future studies should investigate how these axes interact with the CXCL12-CXCR4 pathway and whether they are differentially regulated across OLP subtypes.

In conclusion, as the organism ages and cellular senescence progresses, senescent mesenchymal cells and their secreted signalling molecules modulate CD8^+^ T cells and NK cells, both of which are intricately linked to the pathology of OLP. This interaction ultimately promotes the pathological alterations characteristic of OLP. It is indisputable that OLP has a multifactorial aetiology. Recent research has increasingly recognised and investigated the effects of age-related autoimmune factors, particularly the alterations in adaptive immune cells, including T cells and NK cells, on the pathogenesis of OLP.

#### Behçet's disease (BD)

BD, also known as Behçet's syndrome, is a chronic, rare multisystem disorder characterised by autoimmune and inflammatory features. It manifests with ocular involvement, recurrent genital and oral ulcers, skin symptoms, arthritis and potential involvement of the nervous system, gastrointestinal tract and vasculature [68]. BD is classified as an autoimmune disorder characterised by infiltration of lymphocytes and neutrophils into target organs, elevated immunoglobulin secretion, formation of immune complexes and production of acute-phase proteins. These immune dysregulations contribute to the pathogenesis of BD [4]. Evaluation of cytokine profiles in patients with BD reveals a predominance of T helper 1 (Th1) cytokines, which may contribute to the activation of neutrophils and endothelial cells [69]. While the precise pathogenesis of BD remains unclear, research suggests that immune dysregulation may underlie its development. A prevailing hypothesis holds that the inflammatory response is initiated by infectious agents or autoantigens in genetically susceptible individuals and sustained by both innate and adaptive immune mechanisms. Moreover, a growing body of evidence implicates T helper 17 (Th17) cells as significant contributors to the pathophysiology of BD [70].

Although BD was historically considered a Th1-mediated disorder, a growing body of evidence suggests that both Th1 and Th17 pathways are implicated in its pathogenesis, each contributing via distinct proinflammatory cytokines [70]. Research has established that Th17 cells constitute a distinct subset of helper T cells, independent of the Th1 and Th2 lineages and implicated in a variety of autoimmune and inflammatory diseases [71–73]. Th17 cells modulate inflammatory responses by inducing various cytokines, including IL-17, IL-21, IL-22 and IL-26 [74]. In active BD, both the proportion of circulating Th17 cells and their capacity to produce the signature Th17 cytokine IL-17 are significantly elevated. Conversely, Th17 levels are notably reduced during the remission phase [75,76]. Nonetheless, emerging evidence suggests that the balance between Th17/Th1 and Th17/Treg is a critical regulatory factor in the inflammatory activity of BD [77–80]. In the context of BD-associated inflammation, Treg cells can transdifferentiate into Th17 cells in response to cytokines such as IL-1 or IL-2 [76,80]. Conversely, the decline in Th17 levels during the remission phase of BD is believed to stem from the transdifferentiation of Th17 cells into Treg cells [76,81]. Studies have demonstrated that IL-21 stimulation of Naïve CD4^+^ T cells promotes the differentiation of both Th17 and Th1 subsets while concurrently decreasing the proportion of Treg cells in peripheral circulation. Inhibition of IL-21 has been shown to restore Th17/Treg homoeostasis effectively.

Research has identified a distinct subset of T cells known as Tγδ cells, which express γδ chains [82] on their surface and serve as the initial line of defence against microbial infections. Tγδ cells infiltrate sites of microbial presence by recognising microbial products present in the oral mucosal ulcers of patients with BD. Additionally, they influence adaptive immune responses by secreting IFN-*γ* or IL-4 [83]. Experimental models have shown that Tγδ cells can induce the differentiation of Th1 and Th17 cell subsets [84]. In vitro studies have found that antigens derived from *Streptococcus*, *Escherichia coli* and *Staphylococcus aureus*, as well as non-peptide antigens, can robustly activate γδ T cells in patients with BD. This suggests that the T lymphocytes of BD patients may exhibit generalised hyperactivity to bacterial antigens rather than simply adapting to specific antigenic signals. T cells mediate neutrophil activation by producing proinflammatory cytokines, particularly IL-17, which is secreted by Th17 cells [85]. There is substantial evidence indicating that TNF-*α* plays a central role in immune responses [86,87]. T cells can produce this inflammatory cytokine and act on them to promote activation and proliferation. Additionally, TNF-*α* can induce apoptosis in highly activated effector T cells and exert bidirectional effects on Treg cells, downregulating their suppressive capacity while promoting their proliferation [88].

BD is characterised as a recurrent multisystem inflammatory disorder that typically emerges in individuals during their third to fourth decades of life, with no discernible gender bias. As age increases, disease activity generally declines in younger, reproductively active populations after experiencing significant and recurrent exacerbations [40]. Regarding the relationship between genetic and immunological factors in BD, extensive research has delineated the role of hereditary influences, with the HLA-B*51 allele identified as a prominent risk factor for the disease [89,90]. HLA-B*51 plays a crucial role in peptide presentation to CD8^+^ T cells, implicating these cells in the pathogenesis of BD. CD8^+^ T cells secrete a variety of cytokines, including IL-17, IL-8 and granulocyte-macrophage colony-stimulating factor (GM-CSF), which enhance neutrophil activation and facilitate interactions between innate and adaptive immunity, contributing to the immunopathogenic mechanisms of BD [91]. Additionally, research has demonstrated that immune-mediated networks contribute significantly to the inflammatory cascade. The pathogenesis of BD has been classified as an intersection of autoimmune and autoinflammatory syndromes [71]. On the one hand, observations indicate that BD may exhibit inflammatory characteristics. Unlike other autoimmune disorders, the pathogenesis of BD is not associated with specific autoantibodies, although it shares features with certain autoinflammatory conditions. Nonetheless, evidence also supports the notion that BD possesses autoimmune features, particularly in its association with class I MHC (HLA-B51). The activation of the adaptive immune system is a principal driver of inflammatory processes in autoimmune diseases, with Th1 and Th17 cells playing pivotal roles in the pathogenesis of BD [92]. Th17 cells are involved in the pathological induction and proliferation of autoimmunity, while Treg cells play a crucial role in suppressing autoimmunity and fostering tolerance to self-antigens [93]. The balance between Th17 and Treg cells provides a foundational understanding for elucidating and regulating the immunological mechanisms of BD, which is primarily characterised by immune responses mediated by Th1 and Th17 cells.

BD is known to be an aetiologically undefined systemic vasculitis, with a more active and severe disease course in the early years of onset. One of the most intriguing features of BD is its tendency to progress to low activity and remission over time [94]. In innate immunity, the antimicrobial activity of neutrophils diminishes with advancing age over time [94]. Furthermore, with advancing age, proinflammatory cytokines increase, paralleled by an increase in anti-inflammatory cytokines [95]. With aging, the proliferative capacity and functional characteristics of NK cells also decline [96,97]. In adaptive immunity, TCR involvement in the proliferative response of senescent T cells is also compromised. Additionally, the rate of non-Treg T lymphocytes converting into Tregs increases with age, resulting in a rise in the population of memory Tregs [98]. The age-associated reduction in the IL-17/Treg ratio is closely linked to increased IL-10, thereby influencing the delicate balance between proinflammatory and anti-inflammatory immune responses [99]. Studies indicate that age-standardised mortality rates, especially among younger males (ages 14–24 and 25–34), have risen, whereas older males (ages 35–50) demonstrate normal life expectancy. Longitudinal comparisons across the first 7, 14 and 20 years of the disease reveal a significant decline in mortality rates [94]. T-cell exhaustion is not an optimal condition in chronic viral infections and cancer; however, it is more desirable in autoimmune and inflammatory diseases. Immune checkpoint inhibitors are employed to mitigate T-cell exhaustion in certain cancer types. Conversely, these agents may precipitate the development of vasculitides or autoimmune diseases [100]. Consequently, T-cell exhaustion may confer distinct advantages in the context of autoimmune diseases. Although current research has not conclusively determined whether T-cell exhaustion manifests over time in patients with BD, this phenomenon could potentially facilitate the amelioration of BD symptoms. It is tempting to speculate that T-cell exhaustion potentially arising from repeated exogenous and/or endogenous antigen stimulation or as part of immunosenescence might contribute to the progressive improvement observed in some patients with BD over time. However, this hypothesis requires direct experimental validation, and the relative contributions of T-cell exhaustion versus other age-related immune changes (e.g. reduced neutrophil activity, diminished NETosis, decreased ROS production) remain to be delineated. [94]. The relevant literature indicates that, from a clinical standpoint, late-onset BD shares many characteristics with early-onset BD. However, in terms of disease activity, late-onset BD has been observed to exhibit lower activity levels. This finding may suggest that reduced disease activity is associated with age-related alterations in the immune system [94].

Further studies indicate that older patients with BD exhibit lower disease activity, likely due to immune changes associated with immunosenescence, chronic inflammation and age-related hormonal variations (Figure 3). With age, decreased disease activity and improved prognosis in male BD patients may be linked to age-associated changes, including reduced neutrophil activity, diminished NETosis and reduced reactive oxygen species (ROS) production. Additionally, T-cell exhaustion potentially arising from repeated exogenous and/or endogenous antigen stimulation or immune aging may play a supplementary role in the pathophysiological mechanisms of BD [94].

**Figure 3.**
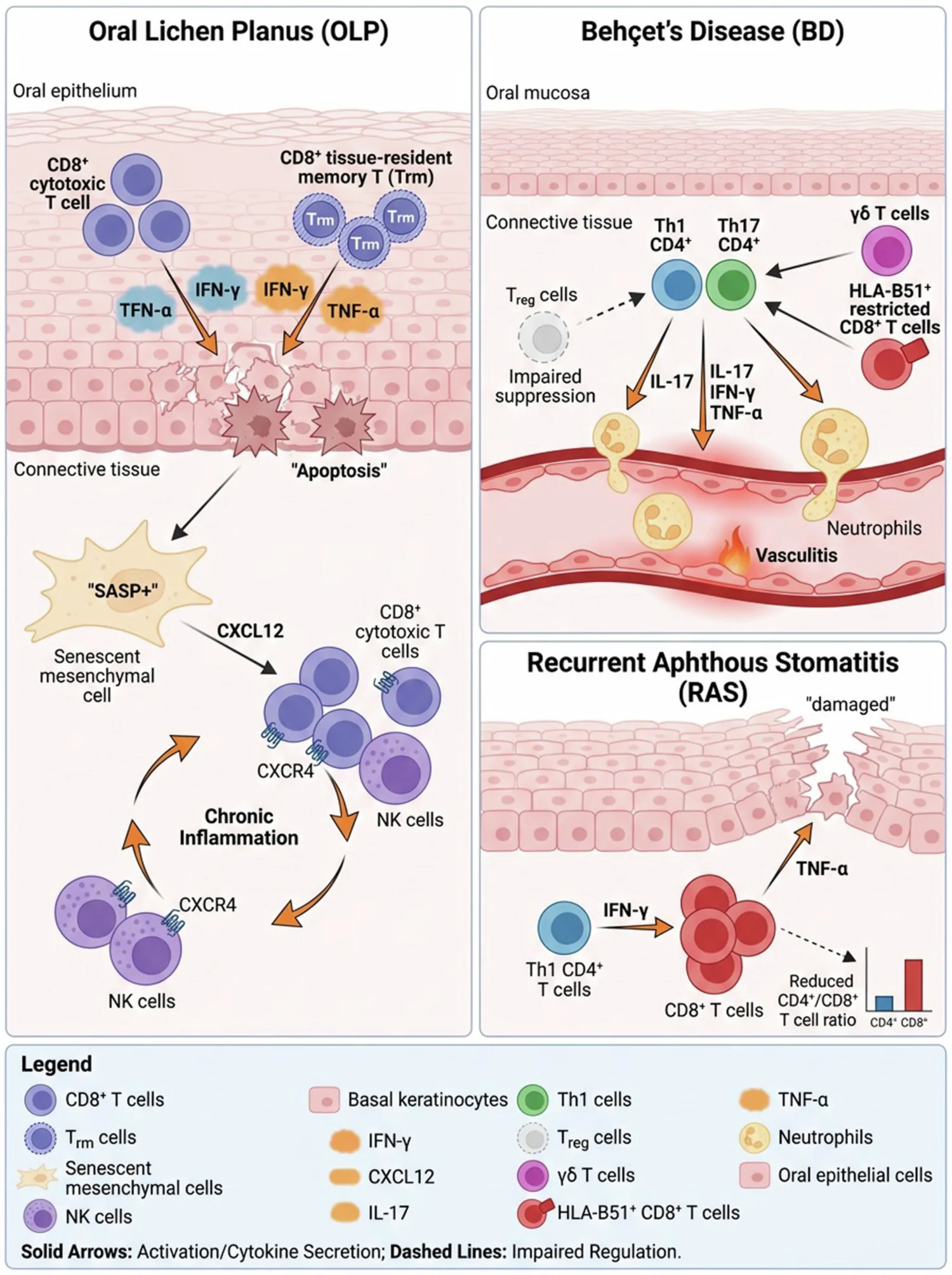
Key T-cell subset alterations and cytokine networks in oral lichen plaques, Behçet's disease and recurrent aminoglycous stomatitis: Left panel (oral lichen planus—OLP): CD8⁺ cytotoxic T cells and CD8⁺ tissue-resident memory T (Trm) cells infiltrate the oral epithelium and secrete IFN-*γ* and TNF-*α*, leading to basal keratinocyte apoptosis. Senescent mesenchymal cells with a senescence-associated secretory phenotype (SASP) further activate and recruit CD8⁺ T cells and NK cells via the CXCL12-CXCR4 axis, thereby perpetuating chronic inflammation. Middle panel (Behçet's disease—BD): An imbalance between Th1/Th17 (increased) and Treg (decreased) populations promotes the production of IL-17, IFN-*γ* and TNF-*α*, which activate neutrophils and vascular endothelium, causing vasculitis. γδ T cells and HLA-B51-restricted CD8⁺ T cells also contribute to the inflammatory cascade. Right panel (Recurrent aphthous stomatitis—RAS): A Th1-skewed immune response and CD8⁺ T-cell activation lead to elevated TNF-*α* levels, which directly damage oral epithelial cells. A reduced CD4⁺/CD8⁺ T-cell ratio further compromises immune regulation. It is worth noting that the pathways depicted are schematic representations based on current evidence and hypotheses. Direct causal relationships between specific T-cell subsets and disease outcomes require further experimental validation.

## Recurrent aphthous stomatitis (RAS)

RAS, also known as recurrent aphthous ulcers (RAU) or recurrent oral ulcers (ROU), is a chronic, inflammatory, ulcerative disease of the oral mucosa [101]. RAS is characterised by its periodicity, recurrence and self-limiting nature, often manifesting as painful ulcers with significant burning sensations. The aetiology and pathogenesis of RAS remain poorly understood; however, contemporary research suggests that genetic mechanisms mediating both innate and adaptive immune responses are instrumental in the disease's progression. Evidence indicates that immune function in RAS patients is disrupted by specific yet-to-be-identified triggering factors, potentially including viral and bacterial antigens or psychosocial stressors [101]. In individuals with RAS, both innate and adaptive immune responses, including humoral and cellular components, are dysregulated. This is evidenced by the reactivation and heightened reactivity of neutrophils, elevated concentrations of complement components, and an increased abundance of NK cells and B lymphocytes. Additionally, there is a significant perturbation in the proportions and numbers of CD25^+^ T cells and TCR γδ T cells in peripheral blood [1,2]. Moreover, numerous studies suggest that Th1-type immune responses play a crucial role in the pathogenesis of RAS [102–104].

Extensive research indicates that immune factors are among the most significant pathogenic mechanisms underlying RAS, particularly cellular immune responses. Studies on the immunological aetiology of RAS primarily focus on cellular immunity, revealing a decline in cellular immune function and an imbalance in T-lymphocyte subsets in affected individuals. In patients with RAS, complement components and immunoglobulins may remain within normal ranges, while circulating immune complexes and antibody-dependent cytotoxic cells increase in peripheral blood. Immunofluorescence studies suggest the presence of autoantigens in the cytoplasm of keratinocytes within pathological sections of RAS, as well as a fluorescent effect at the basement membrane or the presence of circulating antibodies against oral mucosa. However, the absence of antinuclear antibodies, commonly found in autoimmune diseases, indicates that humoral immunity and autoimmune responses are likely only one of several contributing factors in the pathogenesis of RAS. Evidence suggests that chronic inflammation is fundamentally rooted in immunological mechanisms, with T cell-mediated immunity playing a pivotal role in the pathogenesis of RAS. A marked imbalance between CD4^+^ and CD8^+^ T lymphocytes, characterised by a reduced CD4+/CD8+ ratio, is commonly observed. Furthermore, T cells mediate epithelial cell damage by secreting TNF-*α*, and research has demonstrated significantly elevated TNF-*α* levels in the saliva of RAS patients [105].

Considering the clinical characteristics, recurrent aphthae can be classified into three main types: mild aphthae (Mikulicz's aphthae; MiRAS), severe aphthae (Sutton's aphthae; MaRAS) and herpetiform aphthae (HeRAS) [106,107]. The second decade of life is recognised as a critical period for RAS incidence, with initial episodes often manifesting in childhood or later life, notably, as with other disorders potentially characterised by autoimmune mechanisms, an earlier onset of RAS does not inherently predict a worse prognosis [108]. Epidemiological observations indicate that MaRAS is more prevalent among younger patients, whereas HeRAS is most common among individuals in their thirties [109–111]. The frequency of RAS typically decreases with age [112,113]. Compared with younger patients, older individuals exhibit a reduced incidence of RAS, which may be partly attributed to age-dependent changes in both innate and adaptive components of the immune system, commonly referred to as ‘immune senescence’ and ‘inflammaging’ [114,115]. In the geriatric population, there is a notable reduction in the chemotactic and phagocytic functions of neutrophils, coupled with a diminished ratio of naïve T cells relative to their memory counterparts, leading to an overall decrease in cellular populations [116,117]. In addition to alterations in the composition of immune cell populations, there are significant changes in cytokine production and reactivity, accompanied by reduced proliferative responses, defects in signal transduction and diminished capacity for antigen recognition [116,118]. The lower prevalence of RAS in the elderly population is likely multifactorial. However, the age-related increase in peripheral CD4⁺CD25ʰⁱᵍʰFOXP3⁺ regulatory T cells may be one contributing factor, potentially by suppressing autoreactive T-cell responses. Other age-associated changes—including reduced neutrophil chemotaxis and phagocytosis, diminished naïve-to-memory T-cell ratios and altered cytokine production profiles—likely also play important roles [119]. This immunological shift could also be relevant to the pathophysiology of RAS. A comparative summary of the T-cell subset alterations, cytokine profiles and pathological mechanisms for OLP, BD and RAS is presented in Table 1 and Figure 4.

**Figure 4.**
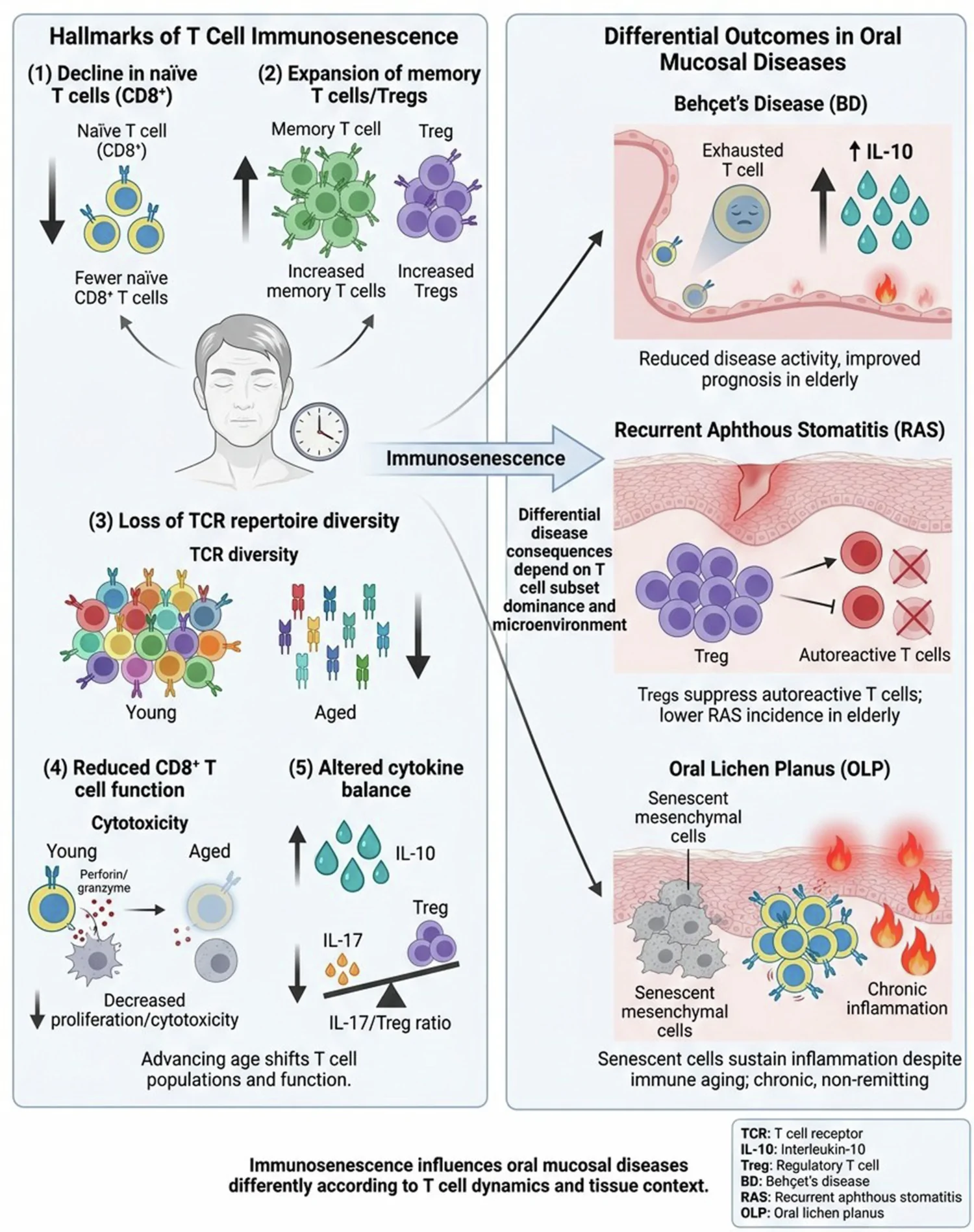
Impact of immunosenescence on T-cell dynamics and oral mucosal disease outcomes: Left panel—Hallmarks of T-cell immunosenescence: Advancing age leads to (1) decline in naïve T cells (particularly CD8⁺), (2) expansion of memory T cells and regulatory T cells (Tregs), (3) loss of T-cell receptor (TCR) repertoire diversity, (4) reduced CD8⁺ T-cell proliferative and cytotoxic capacity and (5) altered cytokine balance (e.g. increased IL-10, decreased IL-17/Treg ratio). It is worth noting that this figure illustrates proposed relationships between immunosenescence and disease outcomes based on available evidence. The differential effects on BD, RAS and OLP represent working hypotheses that warrant further investigation. Right panel—Differential disease consequences: Behçet's disease (BD): Immunosenescence-associated T-cell exhaustion and increased anti-inflammatory cytokines (e.g. IL-10) correlate with reduced disease activity and improved prognosis in older patients. Recurrent aphthous stomatitis (RAS): Age-related expansion of Tregs may suppress autoreactive T-cell responses, contributing to the lower incidence of RAS in older people. Oral lichen planus (OLP): Persistent accumulation of senescent mesenchymal cells sustains CD8⁺ T-cell activation, leading to chronic, non-remitting inflammation despite overall immune aging.

**Table 1.** Role of T cells in major oral mucosal diseases.

Immune cell/subset	Oral lichen planus	Behçet's disease	Recurrent aphthous stomatitis
CD8^+^ cytotoxic T cells	Accumulate at the epithelial–connective tissue junction; induce apoptosis of basal keratinocytes via IFN-*γ* and cytotoxic mediators [52–54]; central drivers of epithelial damage.	Increased cytotoxic CD8^+^ T cells contribute to vascular inflammation and tissue injury, which are associated with HLA-B*51 antigen presentation [90,91].	Elevated CD8^+^ T-cell activity contributes to epithelial damage and ulcer formation [106].
CD8^+^ tissue-resident memory T cells	Highly enriched in erosive OLP; secrete IFN-*γ*, TNF-*α*, IL-17 [52–54]; promote lesion persistence and recurrence [56].	Not clearly defined; tissue-resident cytotoxic responses may participate in chronic inflammation.	Currently unclear; potential role in local immune memory remains speculative.
CD4^+^ Th1 cells	Secrete IL-2 and IFN-*γ* to support CD8^+^ T-cell activation and keratinocyte apoptosis [55].	Predominant Th1 cytokine profile; promotes neutrophil activation and endothelial inflammation [70].	Th1-skewed immune response contributes to chronic inflammation [103–105].
CD4^+^ Th17 cells	Contribute to local inflammation and epithelial injury; an increase in aging gingival tissue.	Expanded in active disease; IL-17 drives inflammation; imbalance with Treg cells correlates with disease activity [71].	Increased Th17 responses promote ulcer persistence and inflammation.
Regulatory T cells	Functional alterations may impair immune tolerance, contributing to chronic inflammation.	Reduced number and function during active disease; Th17/Treg imbalance is a key pathogenic factor [78–81].	An age-related increase in Treg cells may explain the reduced disease frequency in elderly patients [115,116].
γδ T cells	Limited evidence; potential role in epithelial immune surveillance.	Activated by microbial antigens in oral ulcers; promotes Th1 and Th17 differentiation [85].	Altered γδ T-cell proportions observed in peripheral blood [1,2].
T-cell senescence/immunosenescence	Senescent T cells and SASP-driven signalling exacerbate epithelial damage and chronic inflammation [64].	T-cell exhaustion and immunosenescence may contribute to reduced disease activity with age [95].	Immune aging is associated with decreased incidence and severity [115,116].

## Therapeutic implications: targeting T-cell senescence and immunosenescence

The emerging understanding of T-cell senescence and immunosenescence in oral mucosal diseases opens new avenues for therapeutic intervention. Several strategies are currently under investigation:

Senolytic agents that selectively eliminate senescent cells have shown promise in preclinical models of autoimmune and inflammatory diseases. By clearing senescent mesenchymal cells that drive CD8⁺ T-cell recruitment via the CXCL12-CXCR4 axis in OLP, senolytics could potentially attenuate chronic inflammation. However, their application in oral mucosal diseases remains experimental (Table 2).

**Table 2.** Comparison of T‑cell‑related immune features among oral lichen planus (OLP), Behçet's disease (BD) and recurrent aphthous stomatitis (RAS).

Feature	OLP	BD	Recurrent Aphthous Stomatitis (RAS)
Major T-cell subsets involved	CD8⁺ cytotoxic T cells, CD8⁺ tissue-resident memory T (Trm) cells	Th1, Th17, γδ T cells, CD8⁺ T cells	Th1, CD8⁺ cytotoxic T cells
Key cytokines	IFN-*γ*, TNF-*α*, IL-17	IL-17, IFN-*γ*, TNF-*α*, IL-21	TNF-*α*, IFN-*γ*, IL-2
Treg changes	Functional impairment possible	Decreased numbers and suppressive function during active disease	Age-related increase in Tregs
Evidence for immunosenescence	Senescent mesenchymal cells (SASP) recruit CD8⁺ T and NK cells via the CXCL12–CXCR4 axis; senescent T cells aggravate epithelial damage.	Telomere dysfunction promotes CD4⁺ T-cell senescence; T-cell exhaustion may attenuate disease activity with age.	Lower incidence and severity in older people, partly attributable to increased Tregs and reduced effector responses.
Main pathological mechanism	CD8⁺ T-cell-mediated apoptosis of basal keratinocytes; chronic inflammation sustained by Trm and SASP	Th1/Th17 polarisation with Treg deficiency; vascular inflammation; neutrophil activation	Th1-skewed response and CD8⁺ T-cell activation causing epithelial injury; reduced CD4⁺/CD8⁺ ratio
Age-related trend	Chronic, non-remitting inflammation persists despite overall immunosenescence.	Disease activity and severity tend to decrease with age	Frequency and severity generally decline with age

Modulation of the senescence-associated secretory phenotype (SASP) represents another approach. Targeting specific SASP components such as IL-6, IL-8, or TNF-*α* with neutralising antibodies or small-molecule inhibitors could reduce the proinflammatory milieu that sustains T-cell activation and tissue damage [120].

Restoring Th17/Treg balance is particularly relevant for BD, where Th17 expansion and Treg deficiency drive inflammation. Therapeutic strategies that promote Treg differentiation or inhibit Th17 polarisation, such as IL-21 blockade or low-dose IL-2 therapy, may offer benefit.

Immune checkpoint modulation presents a double-edged sword. While checkpoint inhibitors (e.g. anti-PD-1/PD-L1) are used to reverse T-cell exhaustion in cancer, they can precipitate autoimmune-like adverse events. Conversely, in autoimmune diseases like BD, promoting T-cell exhaustion (rather than reversing it) might be therapeutically desirable.

Importantly, translating these strategies into clinical practice faces substantial hurdles, including the lack of oral mucosa-specific delivery systems, potential off-target effects on protective immunity and the need for biomarkers to identify patients most likely to benefit. Nevertheless, targeting T-cell senescence represents a promising frontier for the management of chronic oral mucosal inflammatory diseases.

## Conclusion

The oral mucosa functions as a dynamic barrier, continuously exposed to a range of environmental stimuli. An expanding body of clinical evidence and research highlights the considerable influence of autoimmune factors on oral mucosal diseases, particularly regarding T-cell alterations induced by immunosenescence. However, further investigation through research and clinical trials is imperative to fully elucidate the complex mechanisms underlying the interplay between T-cell changes and the pathogenesis of oral mucosal diseases.

## Data Availability

Not applicable.
